# Heterochromatic Genes Undergo Epigenetic Changes and Escape Silencing in Immunodeficiency, Centromeric Instability, Facial Anomalies (ICF) Syndrome

**DOI:** 10.1371/journal.pone.0019464

**Published:** 2011-04-29

**Authors:** Marie-Elisabeth Brun, Erica Lana, Isabelle Rivals, Gérard Lefranc, Pierre Sarda, Mireille Claustres, André Mégarbané, Albertina De Sario

**Affiliations:** 1 CNRS UPR 1142, Montpellier, France; 2 INSERM U827, Montpellier, France; 3 Université Montpellier 1, Montpellier, France; 4 ESPCI ParisTech, Paris, France; 5 Université Montpellier 2, Montpellier, France; 6 CHRU, Montpellier, France; 7 Unité de Génétique Médicale and Laboratoire Associé INSERM à l’UMR S910, Faculty of Medicine, Saint Joseph University, Beirut, Lebanon; 8 Institut Jérôme Lejeune, Paris, France; Florida State University, United States of America

## Abstract

Immunodeficiency, Centromeric Instability, Facial Anomalies (ICF) syndrome is a rare autosomal recessive disorder that is characterized by a marked immunodeficiency, severe hypomethylation of the classical satellites 2 and 3 associated with disruption of constitutive heterochromatin, and facial anomalies. Sixty percent of ICF patients have mutations in the *DNMT3B* (*DNA methyltransferase 3B*) gene, encoding a *de novo* DNA methyltransferase.

In the present study, we have shown that, in ICF lymphoblasts and peripheral blood, juxtacentromeric heterochromatic genes undergo dramatic changes in DNA methylation, indicating that they are *bona fide* targets of the DNMT3B protein. DNA methylation in heterochromatic genes dropped from about 80% in normal cells to approximately 30% in ICF cells. Hypomethylation was observed in five ICF patients and was associated with activation of these silent genes. Although DNA hypomethylation occurred in all the analyzed heterochromatic genes and in all the ICF patients, gene expression was restricted to some genes, every patient having his own group of activated genes. Histone modifications were preserved in ICF patients. Heterochromatic genes were associated with histone modifications that are typical of inactive chromatin: they had low acetylation on H3 and H4 histones and were slightly enriched in H3K9Me_3_, both in ICF and controls. This was also the case for those heterochromatic genes that escaped silencing. This finding suggests that gene activation was not generalized to all the cells, but rather was restricted to a clonal cell population that may contribute to the phenotypic variability observed in ICF syndrome. A slight increase in H3K27 monomethylation was observed both in heterochromatin and active euchromatin in ICF patients; however, no correlation between this modification and activation of heterochromatic genes was found.

## Introduction

ICF (Immunodeficiency, Centromeric Instability, and Facial Anomalies; OMIM #242860), is a rare autosomal recessive disorder. Up to now, less than fifty cases have been reported. Most of the ICF patients were born from consanguineous marriages and about 60% had mutations in the *DNMT3B* (*DNA methyltransferase 3B*) gene that maps to the 20q11.2 region [1]–[3]. The DNMT3B enzyme catalytically methylates cytosines in CpG pairs, which are later bound by repressor complexes.

Several mutations have been described in ICF syndrome, most of them (79%) lying in the gene portion encoding the C-terminal catalytic domain. These mutations induce a decrease of the enzymatic activity. The less frequent N-terminal mutations are mostly nonsense mutations that arise as compound heterozygous mutations. Forty percent of clinically diagnosed ICF patients do not carry any mutation of this gene, suggesting mutational heterogeneity or involvement of other genes. Consequently, it has been postulated that there are two types of ICF patients: type 1 patients bearing mutations in the *DNMT3B* gene and type 2 patients without known mutations [4]. In murine models, mutations in the *Dnmt3b* gene negatively affect proper embryonic development [2], [5].

ICF syndrome is characterized by a marked immunodeficiency: patients tend to have low levels of immunoglobulins and may exhibit low levels of B and T cells. The B cell defects associated with agammaglobulinemia or hypogammaglobulinemia in ICF type 1 are characterized by only naïve and no memory B cells in peripheral blood (PB) [6]. Negative selection is impaired and newly generated and immature B cells accumulate in PB due to B cell maturation blockage. Because of chronic respiratory and gastrointestinal infections, many patients die at an early age. Facial anomalies are a heterogeneous trait in ICF syndrome and mainly include hypertelorism, epicanthal folds, abnormally low-set ears, and macroglossia. Stature is often reduced. Centromeric instability is the most typical feature of the disease. The juxtacentromeric heterochromatin of chromosome 1, 9, and 16 is markedly undercondensed and is involved in chromosome rearrangements and multiradiate associations. The instability correlates with a severe hypomethylation of the classical satellites 2 and 3, which are the major components of constitutive heterochromatin. ICF syndrome was the first genetic disease to be associated with a constitutional methylation defect, mainly affecting heterochromatin. The methylation anomalies of ICF syndrome can involve other genomic sequences such as α satellites, the centromeric component of constitutive heterochromatin [7], Alu sequences [8], D4Z4 and NBL2 repeats [9], and imprinted genes [3], [10].

Since it was rather unlikely that hypomethylation of non-coding repetitive sequences accounted for the severe clinical features that characterize ICF patients, several attempts were made in the past to search for genes that lost DNA methylation and, subsequently, became transcriptionally active in these patients. Hansen *et al.* [10] analyzed eleven genes that are normally subject to X inactivation, including SYBL1, that is also present in chromosome Y. In ICF patients, the inactive alleles of these genes were hypomethylated and some of them escaped silencing. Escape from silencing in ICF cells was associated with a marked advance in replication timing from a late to an active X-like pattern [10]. More recently, ICF-specific changes in RNA levels of genes critical for immune function, development, and neurogenesis were identified through the analysis of global expression profiles [11]–[13]. These studies showed that changes in gene expression occurred across the whole genome and in both directions (under- and overexpression) in ICF compared to control lymphoblast cells. In two of these studies, DNA methylation did not change in differently expressed genes [11], [13]; the third study identified subtle but significant changes [12].

In previous reports, we showed that in tumor cells and cancer cell lines juxtacentromeric genes became hypomethylated and escaped silencing [14], [15]. Hypomethylation was very striking (DNA methylation dropped from 80% to less than 40%) and also frequent. In this work, we investigated whether heterochromatic genes escape silencing in ICF cells too.

## Results

### Patient description

Table 1 lists patients and controls analyzed in this study. Four patients (ICF1, ICF2, ICF4, and ICF5) had ICF-specific clinical and karyotypic features. Among them two patients had previously described mutations in the *DNMT3B* gene (type 1 patients) and two siblings had no mutations in the *DNMT3B* gene (type 2 patients). Patient ICF3 presented the cytogenetic rearrangements that are commonly associated with ICF syndrome (chromosome breakages and multiradial configurations); however, he did not suffer from immunodeficiency and had no facial anomalies. Genomic sequencing showed this patient had a missense mutation (K770E) in the *DNMT3B* gene that had never been described before. He was homozygous for this mutation, his parents, who were first cousins, being heterozygous. The missense mutation resulted in an aminoacid change in the catalytic domain. This amino acid is phylogenetically conserved in various animal species.

**Table 1:** List of patients and controls.

	DNMT3BMutations(b)	sex	age (years)	cells (c)
N1	-	F	28	PB
N2	-	M	36	PB
N3	-	F	43	PB
N4	-	M	46	PB
N5	-	M	17	PB
N6	-	M	15	PB
N7	-	F	37	PB
N8	-	M	41	PB
N9	-	M	7	LB
N10	-	F	3	LB
N11	-	M	30	LB
ICF1 (a)	-	M	15	LB, PB
ICF2 (a)	-	M	14	LB
ICF3	K770E	M	13	LB, PB
ICF4	D817G	F	1	LB, PB
ICF5	A603T/STP807ins	F	1	LB

N  =  controls.

(a) brothers.

(b) codon numbers refer to splice isoform GenBank accession no AF156488.

(c) PB, peripheral blood; LB, lymphoblast cells.

### Heterochromatic genes were hypomethylated in ICF cells

**Figure 1:**
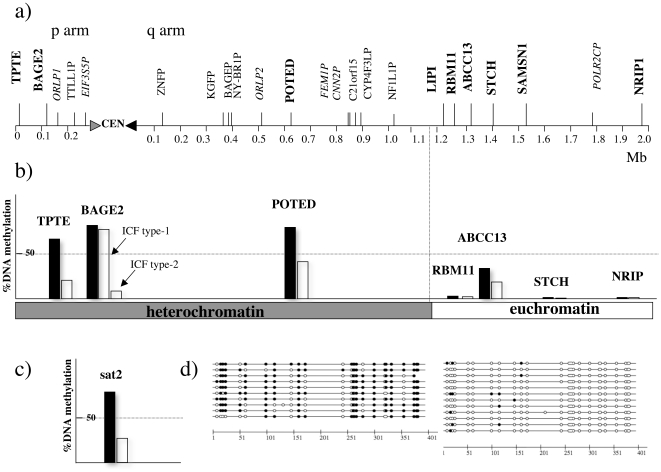
Heterochromatic genes and satellite 2 are hypomethylated in ICF patients. a) Physical map of the 2.3-Mb juxtacentromeric region of human chromosome 21 analyzed in this study. The centromere (cen) is not shown to scale. Genes are bold typed; truncated genes are indicated by plain text and retrotransposed pseudogenes are italicized. b) Histograms represent the percentages of DNA methylation in the analyzed genes either in a pool of controls (black bars) or in a pool of ICF patients (white bars). c) DNA methylation in satellite 2. d) DNA methylation pattern of *TPTE*, a heterochromatic gene, in a control (left) and an ICF patient (right): full circles are 5mCpG and hollow circles are unmethylated CpG.

A large majority of juxtacentromeric regions have been left out from the genome sequencing project as segmental duplications made clone assembly and sequencing troublesome in these chromosome regions. Thus, to determine whether juxtacentromeric heterochromatic genes were hypomethylated in ICF patients, we chose chromosome 21 for which well annotated sequences bridging the chromosome arms with centromeric and juxtacentromeric satellites are available [15]–[17]. A physical map of the 2.3-Mb region that was analyzed in this work is provided in Fig. 1a. Using the CpG island searcher program [18], we scanned the genomic sequence of human chromosome 21 [17] and searched for CpG islands that were associated with the 5’ region of the genes. We analyzed DNA methylation in seven genes mapping to the juxtacentromeric region of human chromosome 21. Three genes (*TPTE*, *BAGE2*, and *POTED*) mapped to the duplication-rich heterochromatic domain and four genes (*RBM11*, *ABCC13*, *STCH,* and *NRIP1*) mapped to the duplication-free euchromatic domain [15], [16]. Two euchromatic genes *LIPI* and *SAMSN1* were excluded from the methylation analysis because the former gene shared a CpG island with *RBM11* (the two genes are head-to-head with the CpG island in the middle) and the latter gene had no associated CpG island. For each gene, we chose a set of nested primers allowing us to amplify a 500-bp DNA stretch in the 5’ CpG island and we analyzed DNA methylation by bisulphite and genomic sequencing in ICF patients and in controls. An example of methylation patterns is shown in Fig. 1d. Since heterochromatic genes belonged to families comprising several loci that have >90% nucleotide sequence identity [16], we selected primers that specifically amplified the gene copy located in chromosome 21. Primer specificity was checked using DNA from a monochromosome hybrid panel. Heterochromatic genes were hypermethylated in controls and hypomethylated in ICF patients (Fig. 1 and Table 2). Hypomethylation was striking (DNA methylation dropped from about 80% in normal cells to approximately 30% in ICF cells) and frequent (present in five analyzed patients). Interestingly, *BAGE2* was hypomethylated only in type 2 patients (χ^2^ test, p-value <0.001). DNA methylation of *BAGE* genes was also analyzed by COBRA as previously described [14]. In this latter case, we measured the global methylation of twelve *BAGE* loci mapped to the juxtacentromeric regions of chromosomes 9, 13, 18, and 21 [19], [20]. *BAGE* loci were hypomethylated in all the ICF patients, regardless of the type, and were hypermethylated in controls. This finding shows that loss of DNA methylation is not restricted to the juxtacentromeric region of chromosome 21, but is common to other chromosomes.

**Table 2:** DNA methylation (%).

	Controls							ICF							
								Type 1			Type 2				
	N9	N10	N2	N6	N7	N4	Total	ICF5	ICF4	ICF3	ICF1	ICF2	Total	p-value	
**Heterochromatin**															
Satellite 2	73.3	71.8	95.2	89.0			81.5	19.1	20.2	69.2	12.9	20.9	27.9	<0.001	
*TPTE*	76.7	64.3	65.8	65.5	62.0	80.8	68.9	11.6	4.7	41.1	38.9	27.3	21.4	<0.001	
*BAGE2*	80.0	75.4	91.7	96.0			84.8	73.2	81.3	83.6	14.9	0	80.3	n.s.	Type1
													8.9	<0.001	Type2
*POTED*	81.0	82.9	83.3	82.1			82.4	41.2	26.1	66.3	30.0	55.5	42.6	<0.001	
**Euchromatin**															
*RBM11*	1.3	1.0	2.9	0.5			1.5	2.2	0.4	2.0	0.6	0	1.1	n.s.	
*ABCC13*	36.4	33.3	35.8	35.4			35.2	21.6	23.4	25.3	14.4	12.2	19.3	<0.001	
*STCH*	0.7	1.5	0	0			0.7	0.4	0.5	0.5	0	0.4	0.4	n.s.	
*NRIP1*		0.3	2	0			0.7	0	1.7	0.7	1.7	0	0.8	n.s.	

n.s., not significant.

Genes located in the euchromatic duplication-free domain were hypomethylated both in ICF patients and controls. One exception was represented by the *ABCC13* gene, which was slightly methylated in controls (35.2% 5mCpG) and significantly hypomethylated in ICF patients (17.5% 5mCpG) (χ^2^test, p-value <0.001).

To achieve our analysis, we analyzed DNA methylation of satellite 2 using the bisulphite and genomic sequencing method. Satellite 2 was much less methylated in ICF patients than in controls (χ^2^test, p-value <0.001) (Fig. 1c).

In patient ICF3, satellite 2 was as methylated as in controls; in contrast, two heterochromatic genes (*TPTE* and *POTED*) and the normally methylated euchromatic gene *ABCC13* had a significant, but less striking loss of DNA methylation compared to the other patients (Table 2).

### Heterochromatic genes escaped silencing in ICF cells

**Figure 2:**
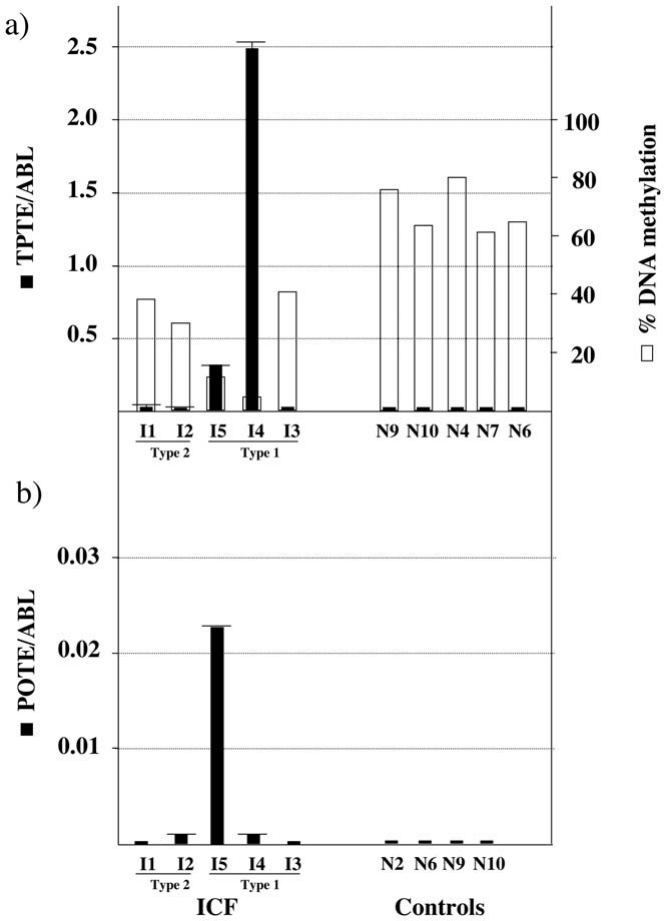
Heterochromatic genes escape silencing in ICF patients. Black bars represent gene expression of two heterochromatic gene families that were expressed in ICF patients: *TPTE* (a) and *POTE* (b). The *ABL* gene was used to normalize cDNA amounts. I, ICF patients; N, controls. Type 1, 2 are ICF patients with and without known mutations in the *DNMT3B* gene, respectively. For *TPTE* (a) we superposed the percentage of DNA methylation (white bars).

Afterwards, we decided to investigate whether DNA hypomethylation was associated with the activation of heterochromatic genes in ICF patients. Gene expression was first analyzed by reverse transcription and standard PCR on total RNA in five ICF patients and eleven controls. Either *TPTE,* or *POTE,* or both genes were expressed in four ICF patients, but were silent in controls. No heterochromatic genes were expressed by patient ICF3. The *BAGE* genes were expressed neither in ICF patients nor in controls. The expression levels of *TPTE* and *POTE*, which were specifically activated in ICF cells, was measured by real-time PCR (Fig. 2). The level of expression of *TPTE* was correlated with the degree of hypomethylation (Fig. 2a). Primers used for the expression analysis of heterochromatic genes were not specific for the gene copies located on chromosome 21. Thus, to determine which *POTE* and which *TPTE* were expressed by the ICF cells, we cloned and sequenced the RT-PCR products. The ICF cells expressed *POTEF* from chromosome 2, *POTEG* from chromosome 14, but they did not express *POTED* from chromosome 21. ICF patients also expressed *TPTE* from chromosome 21 and *TPTE2* from chromosome 13[21]. Overall, every patient had his own group of activated genes and activation of heterochromatic genes was not restricted to chromosome 21, but occurred in several chromosomes (Table 3). Genes located in the euchromatic duplication-free domain had the same expression pattern in ICF patients as in controls: *SAMSN-1* and *STCH* were strongly expressed, *NRIP1* was weakly expressed, and *LIPI*, *RBM11*, and *ABCC13* were silent.

**Table 3:** Heterochromatic genes activated in ICF cells.

ICF1		*TPTE2*	
ICF2		*TPTE2*	*POTEG*
ICF4	*TPTE*		*POTEF*
ICF5	*TPTE*	*TPTE2*	*POTEF*

### Activation of heterochromatic genes was not associated with chromatin changes in ICF cells

**Figure 3:**
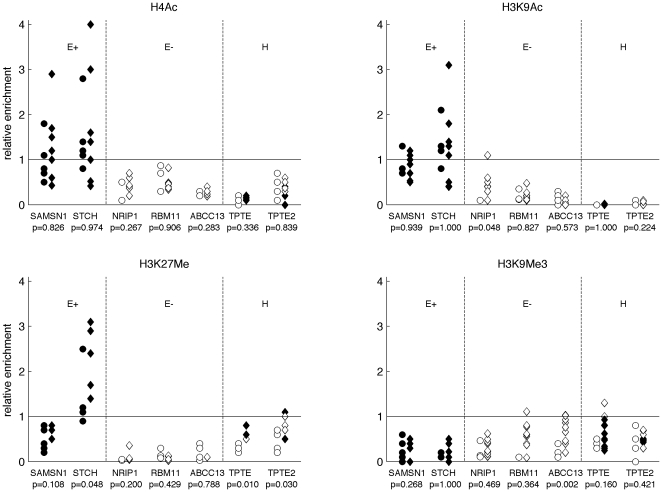
Relative enrichment of histone modifications in expressed euchromatic (E+), silent euchromatic (E–), and heterochromatic (H) genes. Symbols represent single experiments either in an ICF (diamond) or in a control (circle) cell line. Filled markers denote expressed genes. The significance of the differences between ICF patients and controls was evaluated for each modification and for each gene using Wilcoxon’s rank sum test (p).

Histone modifications were analyzed in three ICF (ICF3, ICF4, and ICF5) and three control (N9, N10, and N11) lymphoblast cell lines. Two patients (ICF1 and ICF2) could not be analyzed, because their cell lines were lost during the course of this project. ChIP (Chromatin Immunoprecipitation) experiments were done using antibodies against four histone isoforms (H3K9Me_3_, H3K27Me, H3K9Ac, and H4Ac) (Fig. 3).

Active and silent heterochromatic genes had similar histone modifications. We analyzed *TPTE* and *TPTE2,* but not *POTE* genes. In this gene family, given the presence of thirteen copies dispersed on eight chromosomes [22], we failed to design chromosome-specific primers for real-time PCR.

Then, we compared histone modifications in ICF patients versus controls in the whole set of juxtacentromeric genes. Histone acetylation and H3K9 trimethylation were similar in ICF patients and in controls; in contrast, monomethylation of H3K27 in three genes (*TPTE*, *TPTE2* and *STCH*) was significantly higher in ICF patients than in controls.

Finally, we analyzed histone modifications in the three chromatin domains that are present in the juxtacentromeric region of human chromosome 21: heterochromatin, active euchromatin and silent euchromatin. The heterochromatic genes *TPTE* and *TPTE2* were characterized by very low levels of acetylation both in H4 and in H3K9 histones and had monomethylation of H3K27 and trimethylation of H3K9. In the euchromatic domain, histone modifications correlated with gene expression. *STCH* and *SAMSN-1*, which were highly expressed, were highly acetylated and had low levels of H3K9Me_3_. *STCH* was also highly enriched in H3K27Me. *ABCC13*, *RBM11* and *NRIP1*, three euchromatic genes that were either silent or very weakly expressed, were characterized by very low levels of acetylation and were slightly enriched in H3K9Me_3_. In silent euchromatic genes, H3K27Me was very low.

## Discussion

### Juxtacentromeric heterochromatic genes lose DNA methylation and escape silencing in ICF cells, without apparent chromatin changes

In the present study, we have shown that juxtacentromeric heterochromatic genes lost DNA methylation and escaped silencing in ICF cells. Such a finding was not emphasized by previous cytogenetic analyses of constitutive heterochromatin that detected hypomethylation and chromatin decondensation only in the satellite-rich domains. We analyzed chromosome 21 for which genomic sequences bridging the chromosome arms with centromeric and juxtacentromeric satellites are available [16]–[17]. However, having looked at paralogous genes scattered in various chromosomes, we showed that loss of methylation in juxtacentromeric heterochromatic genes occurred in many chromosomes. Hypomethylation was observed both in type 1 and type 2 patients. Only *BAGE2* was specifically hypomethylated in type 2 ICF patients. A similar finding was reported for α satellite sequences whose hypomethylation was also restricted to type 2 patients [4]. Alpha satellite sequences are located within centromeres and *BAGE2* maps very close to the beginning of α satellite sequences [16]. Centromeric chromatin is structurally and functionally distinct from the flanking juxtacentromeric heterochromatin: it contains blocks of histone H3 nucleosomes interspersed with blocks of CENP-A (an H3 variant) nucleosomes [23]. Centromeric chromatin is also characterized by a pattern of histone modifications that is different from that of the flanking heterochromatin [23]. So, it is tempting to suggest that loss of DNA methylation in ICF patients of type 2 might result from impairment of one of the proteins responsible for the centromeric epigenetic pattern. Interestingly, CENP-C, one of the proteins contributing to centromeric chromatin organization, interacts with DNMT3B and modulates DNA methylation both in centromeric and juxtacentromeric regions [24].

Euchromatic genes were unmethylated both in ICF patients and controls, with the exception of *ABCC13.* In normal cells, this gene was slightly methylated, but less than the heterochromatic genes. The presence of methylation in *ABCC13* is consistent with the notion that it is undergoing a pseudogenization process [16]. *ABCC13* was significantly hypomethylated in ICF cells, showing that hypomethylation occurs also in the euchromatic domain that flanks the constitutive heterochromatin, but is much less striking.

In four ICF patients, heterochromatic genes escaped silencing. Transcription was detected in various paralogs, suggesting that, similar to DNA hypomethylation, gene activation was not restricted to chromosome 21, but occurred in several chromosomes.

Although DNA hypomethylation was generalized to all the heterochromatic genes and to all ICF patients, gene expression was restricted to some genes and every patient had his own group of activated genes. The activated genes differed even in two brothers (ICF1 and ICF2). This finding is consistent with what we previously observed in cancer, where heterochromatic genes became hypomethylated in almost all the tumors, but were expressed only in some of them and every tumor had a specific set of activated genes [14]. The precise mechanism driving transcription of heterochromatic genes in ICF cells is unknown. Clearly, impairment of DNMT3B results in hypomethylation that, however, is not sufficient to allow transcription. Other epigenetic changes might occur, i.e. at the chromatin level. Thus, to better understand gene regulation in human heterochromatic regions, we next analyzed histone modifications by ChIP. Genes located in the euchromatic flanking domain had either active or repressive histone modifications, depending on their expression status. Heterochromatic genes, on the contrary, were associated with histone modifications that were typical of inactive chromatin: they were hypoacetylated and slightly enriched in H3K9Me_3_ and H3K27Me. This was also the case for expressed heterochromatic genes. Since it is unlikely that hypoacetylated genes are transcribed, we suggest that in ICF cells, gene activation is not generalized to all the cells, but rather is restricted to a small percentage of them. Clonal changes in chromatin may result from epigenetic modifications occurring during lymphocyte differentiation. If few cells express heterochromatic genes, transcripts can be detected by RT-PCR; on the other hand, if chromatin changes occur stochastically in a small percentage of cells, ChIP analysis on the whole cell population will not detect them. DNA hypomethylation, in contrast, occurred in all the cells, as the bisulphite and sequencing patterns of different colonies from a same tissue were rather homogeneous (Fig. 1d). It is likely that similar events take place in the inactive X chromosome of ICF cells, where genes subject to inactivation are hypomethylated, escape silencing, but exhibit normal histone modification patterns [25]. Finally, in ICF cells, hypomethylation of subtelomeric regions was associated with aberrant transcription of these sequences, but no information on the chromatin structure was provided [26]. Previous genome-wide transcriptomic analyses identified more than 700 differently expressed genes in ICF patients; however, none of them identified aberrant expression of these heterochromatic genes [12], [13]. One possible explanation is that these previous studies focused on genes expected to be relevant for ICF phenotype, whereas we analyzed specifically heterochromatic genes. In addition, Jin *et al*. [12] selected genes that had a high expression variability between ICF and controls, but not within these two groups of individuals. Yet, the aberrant expression of heterochromatic genes is highly variable within ICF patients. Ehrlich *et al*. [13] did not observe significant changes in differently expressed genes, whereas Jin *et al*. [12] reported significant, but subtle DNA methylation differences in approximately half the analyzed upregulated genes. Our work shows that heterochromatic genes undergo dramatic changes in DNA methylation, indicating that they are *bona fide* targets of the DNMT3B protein.

### H3K27 monomethylation is increased in ICF lymphoblast cell lines

Much less characterized than trimethylation, monomethylation of H3K27 was first described as a repressive mark that is preferentially localized in heterochromatin, but is also found in euchromatic regions [27]. Recent genome-wide analyses have shown that this mark is frequently associated with the promoter of active genes [28]. Our results are consistent with both statements, as we found H3K27 monomethylation in heterochromatin and in active euchromatin, but not in silent euchromatin. Interestingly, H3K27 monomethylation was higher in ICF lymphoblasts than in controls. More patients should be analyzed to corroborate these data. Alteration of this histone mark may be relevant to ICF syndrome, as H3K27 methylation plays an important role during the development of the B lymphoid lineage [29] and ICF patients have immature B cells in the blood [6].

### Aberrant transcription of heterochromatic genes and ICF syndrome

A common feature of heterochromatic genes is their expression pattern mostly restricted to germ cells. *TPTE* and *TPTE2* genes are strongly expressed in testis, in secondary spermatocytes and/or very early spermatids [30]. *POTE* genes are expressed in a few normal tissues (namely testis, prostate, ovary and placenta), in embryonic stem cell lines, and in various cancers [31], [32]. Immunochemistry showed that POTE proteins localized in primary spermatocytes in the seminiferous tubules [33]. The high expression of POTE in spermatocytes suggests a role in spermatogenesis. Finally, *BAGE* genes are exclusively expressed in cancer and testis, encode small putative proteins that have no homology to known domains, and have no orthologs in other mammalian species [19], [20]. Very interestingly, in hypomorphic Dnm3b mutant mice, genes that are normally expressed in testis are aberrantly expressed in somatic embryonic tissues [34]. It is therefore tempting to suggest that the aberrant transcription in somatic tissues of genes that are normally expressed in germ cells may contribute to the phenotypic variability observed in ICF syndrome.

### Incomplete phenotype of an ICF patient

One of the analyzed patients showed an incomplete ICF phenotype (he had neither immunodeficiency, nor the facial anomalies that are typical of ICF patients) and suffered from severe obesity. His karyotype contained multibranched and rearranged chromosomes and, at the molecular level, he was homozygous for a missense mutation resulting in an aminoacid change in the region encoding the catalytic domain of the DNMT3B protein. Loss of DNA methylation was less pronounced than in the other patients and was never associated with activation of expression. It is likely that this previously unreported missense mutation slightly affected the methylation activity of the DNMT3B protein, resulting in genome instability and a less pronounced DNA hypomethylation, without producing the classical ICF phenotype. Other ICF patients having a similar incomplete phenotype may be under-diagnosed.

To conclude, the striking loss of DNA methylation in heterochromatic genes, together with their variable pattern of aberrant expression and the clinical features of the patients involving numerous systems, make ICF syndrome an ideal model to investigate DNA methylation changes and their molecular and pathological consequences.

## Materials and Methods

### Patients and controls

Patients and controls used in this study are listed in Table 1. This study was performed on DNA and RNA extracted from human lymphoblast cell lines and peripheral blood in the context of routine molecular diagnostic service. Prof. André Megarbané received approval from the Institutional Review Board of the Saint Joseph University. Written informed consent to genetic testing was obtained from adult probands or parents in the case of minors after explanation of the nature and its possible implications to the patient and his family.

Three lymphoblast cell lines (ICF5, N10 and N11) were purchased at the Coriell Cell Repositories. The lymphoblast cell line ICF4 was established at the Genethon laboratory (Evry, France). Lymphoblast cell lines ICF1, ICF2, ICF3 and N9 were kindly provided by Dr. Marie-Genevieve Mattei (INSERM UMR 910, Marseille, France).

### DNA and RNA extraction

DNA and RNA were extracted from lymphoblast cell lines and from peripheral blood. DNA was treated with 100 µg/ml proteinase K at 55°C overnight, then extracted with phenol/chloroform, ethanol precipitated and treated with 20 µg/ml RNAse. Total RNA was extracted with Trizol (GIBCO-BRL) according to the manufacturer recommendations.

### Promoter analysis

The chromosome 21 genomic [17] sequence was analyzed with the “CpG island searcher” software [18] to identify gene-associated CpG islands. The following criteria were used: CG% >55% and Observed CpG/Expected CpG >0.65.

### DNA methylation analysis

DNA methylation was analyzed by bisulphite genomic sequencing as described by Frommer *et al*. [35] and by Grunau *et al*. [14] with the following modifications. Three hundred ng of genomic DNA (extracted from peripheral blood when available, otherwise from lymphoblast cell lines) were treated with bisulphite and then purified using the Microcon YM-100 filter device (Millipore) to separate DNA from bisulphite. PCR amplifications were done in two consecutive reactions with a set of nested primers. The PCR program was: 94°C for 2 min; (94°C 1 min, annealing temperature 2 min, 72°C 3 min) for 5 cycles; (94°C 30 sec, annealing temperature 2 min, 72°C 1 min 30 sec) for 24 cycles; 72°C 10 min. Specific primers and annealing temperatures for each gene are listed in Table S1. The PCR products were purified on a low melting agarose gel using the QIAquick gel extraction kit (Qiagen), cloned into a pGEM-T-Easy (Promega) vector and transformed into *E. Coli* JM109 (Promega) cells. Ten colonies per gene per individual were sequenced with T7 and/or SP6 universal primers using a DYEnamic ET Terminator Cycle Sequencing kit (Amersham). The obtained sequences were aligned with the genomic sequence using the GAP4 computer package [36]. Methylation patterns were analyzed with the MethTools software [37]. DNA methylation of *BAGE* genes was also analyzed by COBRA as previously described [14]. Briefly, using bisulphate treated genomic DNA, we amplified simultaneously 12 *BAGE* loci in a single PCR. The obtained product was digested with *Mbo*I and with *Hph*I in two independent assays and methylation patterns were analyzed as previously described [14].

### Expression analysis

*Standard PCR*-Two µg of total RNA (extracted from peripheral blood when available, otherwise from lymphoblast cell lines) were reverse transcribed using the Omniscript kit (Qiagen) according to the manufacturer protocol. Reversed DNA was resuspended in 50 µl of 10 mM Tris. One µl of cDNAs was used for PCR and 10 µl of the reaction were analyzed in ethidium bromide-stained agarose gel. The PCR program was: 94°C 2 min; (94°C 30 sec, 68°C 2 min, 72°C 2 min) for 5 cycles; (94°C 30 sec, annealing temperature 30 sec; 72°C 2 min) for 25 cycles. Specific primers used for expression analysis are listed in Table S2. The housekeeping GAPDH gene was used as control.

*TPTE* and *POTE* RT-PCR products were purified on a low melting agarose gel using the QIAquick gel extraction kit (Qiagen), cloned into a pGEM-T-Easy (Promega) vector, and transformed into *E. Coli* JM109 (Promega) cells. Twelve colonies were sequenced with the T7 universal primer using a DYEnamic ET Terminator Cycle Sequencing kit (Amersham).

*Real-Time PCR*- Two µg of total RNA were reverse transcribed using the QuantiTect Reverse Transcription kit (#205311 Qiagen) according to the manufacturer protocol. Reversed DNA was purified using the Qiaquick (#28704 Qiagen). Expression was quantified using a MyIQ PCR Biorad thermal cycler and the QPCR Rox-&Go Green kit (#EPQON480 MP Biomedicals). PCR reactions were done in 25-µl final volume containing 400 nM of each primer and 1 µl of each template DNA (equivalent to 33 ng of RNA). PCR conditions were the following: 95°C for 15 min, followed by 50 cycles of (95°C 30 s, 60°C 30 s and 72°C 1 min). We used the housekeeping *ABL* gene to normalize cDNA amounts. Primers are listed in Table S2. Each analysis was done in triplicate and assay efficiencies were calculated using cDNA dilution series. To calculate gene expression we used the relative standard curve method.

### Chromatin Immunoprecipitation

ChIP experiments were done in 2–3 biological replicates as previously described [15]. Antibodies were purchased from Upstate (Millipore): anti-trimethylated H3K9 (#07-442), anti-monomethylated H3K27 (#07-448), anti-hyperacetylated histone H4 (penta) (#06-946), and anti-acetylated H3K9 (#07-352).

Real-Time PCR was done in a MyIQ PCR thermal cycler (Biorad) using the QPCR Rox-&Go Green kit (#EPQON480 MP Biomedicals). To amplify genomic DNA we designed primers located in the 3’ end of the CpG islands (Table S2). The euchromatic *GAPDH* locus and a heterochromatic STS (JB 10) served as references to normalize the amount of precipitated DNA (their primers are given in Table S2). For a detailed description of normalization see [15]. Briefly, ratios of precipitated DNA in each gene to that in the reference sequence (either *GAPDH* or STS JB10) were calculated as follows: enrichment factor  =  [ng GENE(B)/ng GAPDH(B)]/[ng GENE(I)/ng GAPDH(I)] with (B) for antibody bound and (I) for input. The unbound fraction of mock-treated chromatin (i.e. precipitation in parallel with the other ChIP but without antibody) was considered as input. DNA immunoprecipitated with anti-trimethylated H3K9 and anti-monomethylated H3K27 was normalized with STS (JB 10); DNA immunoprecipitated with anti-hyperacetylated histone H4 (penta) and anti-acetylated H3K9 was normalized with *GAPDH*.

### Statistical analysis

The analysis of the methylation was performed for each gene using χ^2^ tests at three different levels. First, we checked the homogeneity of the methylation pattern between the cells of each individual on the 2 (5mCpG/CpG) x number of cells contingency table. Aside from a few exceptions, the patients had a homogeneous methylation pattern. Thus, the data corresponding to each individual were pooled, leading to the methylation frequencies for each individual gathered in Table 2. Then, we tested the homogeneity of the ICF patients and that of the healthy controls. The ICF group was always very heterogeneous, whereas the healthy controls showed much more similarity. We nevertheless pooled the data of all ICF patients on one side, and that of all controls on the other side, in order to establish the significance of the methylation difference between ICF and controls, using a χ^2^ test on the corresponding 2×2 contingency table (one degree of freedom). The p-values corresponding to this final test are given in Table 2, as well as the overall methylation frequencies for each group (control and ICF). The significance of the difference between chromatin changes in ICF versus controls was tested separately for each gene and for each histone modification using Wilcoxon’s non-parametric rank sum test.

## Supporting Information

Table S1 Primers used for DNA methylation analysis.
(DOC)

Table S2 Primers used for expression (standard PCR) analysis.
(DOC)
