# Supplementary material for: Heterochromatic Genes Undergo Epigenetic Changes and Escape Silencing in Immunodeficiency, Centromeric Instability, Facial Anomalies (ICF) Syndrome
Source: PLoS One. 2011 Apr 29;6(4):e19464. doi: 10.1371/journal.pone.0019464 (PMC3084872; doi:10.1371/journal.pone.0019464)
Supplement: Table S1 — Primers used for DNA methylation analysis. (DOC) [file pone.0019464.s001.doc]

| **Table S1.** | |  |  |  |  |  |  |  |  |  |
| --- | --- | --- | --- | --- | --- | --- | --- | --- | --- | --- |
| ***Primers used for DNA methylation analysis*** | | | | | | | | | | |
| **Gene** | **primer forward** | **primer reverse** | **nested forward** | **nested reverse** | **°C** | **PCR size (bp)** | **N°CpG** |  |  |  |
| **TPTE** | BS TPTE 122f | BS TPTE 937r | BS TPTE 291f | BS TPTE 735r |  |  |  |  |  |  |
|  | ttgttttttaggttggagtgtagtg | cactaccaattcttactctaccactcc | tgttagttaggatggtttggaattt | cctcctctacaaaatatacctaaac | 52 | 445 | 25 |  |  |  |
| **BAGE2** | BAGE.BS+32f | BAGE.BS+1540r | BAGE-BSF | BAGE-BSR |  |  |  |  |  |  |
|  | tttagaggattaggagaagggggagt | acctaccaattaacattattactaacatta | ggttggagtggtaggagta | ccctaattccccaccacct | 60 | 378 | 12 |  |  |  |
| **POTED** | BS POTE 276f | BS POTE 946r | BS POTE 276f | BS POTE 753r |  |  |  |  |  |  |
|  | tttttttagaggaggttgttagtat | aaaatcaatcccaaccaaaactta | tttttttagaggaggttgttagtat | aaattcaaacccaacaaaaaaatac | 50 | 478 | 42 |  |  |  |
| **RBM11** | rbm11bs-1 | rbm11bs-2 | rbm11bs-3 | rbm11bs-4 |  |  |  |  |  |  |
|  | agttttttgttgtatttatatttatgt | accttatccaaaaaatcacctaatc | gtggttttggagttatgttt | caacctctaaaaaacccttaatc | 58 | 505 | 42 |  |  |  |
| **ABCC13** | abcc13bs-1 | abcc13bs-2 | abcc13bs-3 | abcc13bs-4 |  |  |  |  |  |  |
|  | attggttaggattggaaggtaag | actaaactcctctaattcaaattacac | gggattaaggtgatagttgattttgt | ccctaatctctaatctctactct | 58 | 402 | 29 |  |  |  |
| **STCH** | stchbs53 | stchbs1216 | stchbs357 | stchbs847 |  |  |  |  |  |  |
|  | tagtgttagtaatagagaaaatgtgtt | ccacaaaaaaatcctaaaaatacac | gaattttggatttgattaggggta | tcatctctctaaccatcacaatcc | 60 | 490 | 31 |  |  |  |
| **NRIP1** | RIP140bs-1 | RIP140bs-2 | RIP140bs-3 | RIP140bs-4 |  |  |  |  |  |  |
|  | gtttttttgtttagaattggta | accctactccttttcccttatcttat | tttggatagttttttttggggat | aattaaccctactcctaaatcctac | 45 | 386 | 21 |  |  |  |
| **Sat2** | ST2BS-1 | ST2BS-2 | ST2BS-3 | ST2BS-4 |  |  |  |  |  |  |
|  | ttgaatggaaatgaaaggggttatta | tccaataaattattccattccattcc | tggaaatgaaaggggttattattta | tccattccattaaataattccattc | 60 | 323 | 17 |  |  |  |
|  |  |  |  |  |  |  |  |  |  |  |
|  |  |  |  |  |  |  |  |  |  |  |
|  |  |  |  |  |  |  |  |  |  |  |
|  |  |  |  |  |  |  |  |  |  |  |
|  |  |  |  |  |  |  |  |  |  |  |
