# Supplementary material for: Heterochromatic Genes Undergo Epigenetic Changes and Escape Silencing in Immunodeficiency, Centromeric Instability, Facial Anomalies (ICF) Syndrome
Source: PLoS One. 2011 Apr 29;6(4):e19464. doi: 10.1371/journal.pone.0019464 (PMC3084872; doi:10.1371/journal.pone.0019464)
Supplement: Table S2 — Primers used for expression (standard PCR) analysis. (DOC) [file pone.0019464.s002.doc]

| **Table S2.** | |  |  |  |  |  |  | |  |  |  |
| --- | --- | --- | --- | --- | --- | --- | --- | --- | --- | --- | --- |
| ***Primers used for expression (standard PCR) analysis*** | | | | | | | | | | | |
|  | **primer forward** |  | **primer reverse** |  | **°C** | **PCR size (bp)** | |  |  |  |  |
| **TPTE** | TPTE EXP2 | tccagctacaacatcactggaagt | TPTE EXP1 | tgatgtattcgacggtccacctctg | 60 | 257 | |  |  |  |  |
| **BAGE** | BAGE14 | gtctctggtatctcccgctgag | BAGE19 | ttgctcctgttgagctgccgtctc | 68 | 366 | |  |  |  |  |
|  | BAGE20 | cattttgtaagcactttggagccac | BAGE21 | cactatcagcctcattagaaatctg | 60 | 488 | |  |  |  |  |
| **POTE** | RT.POTE.T445 | tctctggccgtctgtccagataga | RT.POTE.T444 | cactgccaggaagatgaatgtgcg | 60 | 386 | |  |  |  |  |
| **RBM11** | RBM1 | gtgtttgttgggaatttagagg | RBM2 | ctgcacgttaattggtcttcc | 60 | 216 | |  |  |  |  |
| **ABCC13** | PRED6/6 | gattccttctgcactgcgtgtcccatc | PRED6/8 | tcattatgagtgggctagtg | 54 | 228 | |  |  |  |  |
| **STCH** | STCH1 | gccaaagatgaatccttcac | STCH2 | ctccaaattccaacactgtc | 58 | 339 | |  |  |  |  |
| **RIP140** | RIP1 | actgtttattctctgctgc | RIP2 | atacatttgtccagacttgc | 58 | 315 | |  |  |  |  |
| **SAMSN1** | SAMSN6 | gcaggaatacacctcaacac | SAMSN7 | ttaaggagatgtctgagc | 55 | 217 | |  |  |  |  |
| **GAPDH** | G3PDH1 | tgaaggtcggagtcaacggatttggt | G3PDH2 | catgtgggccatgaggtccaccac | 60 | 983 | |  |  |  |  |
|  | | | | | | | | | | | |
| ***Primers used for expression (real-time PCR) analysis*** | | | | | | | | | | | |
| **TPTE** | Q-TPTE-F | ctggcgggagtcatcattg | Q-TPTE-R | gtgtgtgggctttctttcg | 60 | 101 | |  |  |  |  |
| **POTE** | Q-POTE-F | gccatcagaaatagcgtcag | Q-POTE-R | tgagttgttcaatgagcaatg | 58 | 80 | |  |  |  |  |
| **ABL** | Q-ABL-2F | tcttgaactgggcgaatgtc | Q-ABL-2R | aggcgtgctctgtgaaatac | 60 | 134 | |  |  |  |  |
|  | | | | | | | | | | | |
| ***Primers used for CHIP*** | | | | | | | | | | | |
| **TPTE** | QTPTE_chr3 | ttacctatacaccagactgcttcc | QTPTE_chr4 | gaccgacgacacaagacctc | 60 | 104 | |  |  |  |  |
| **TPTE2** | TPIP542F | gggaaaggaatgtcagttagtg | TPIP679R | tgtcctctcatcctcatcag | 60 | 138 | |  |  |  |  |
| **ABCC13** | QABCC_chr5 | cgagcccttgtttcagtagcc | QABCC_chr6 | tcctctcctccagccctacc | 60 | 140 | |  |  |  |  |
| **RBM11** | QRBM_chr3 | ctgttctccgtcaagtagtaagc | QRBM_chr4 | ctgccaaatcatctcaaatctcac | 60 | 160 | |  |  |  |  |
| **NRIP1** | NRIP 13F | aactaacttcccacaggc | NRIP 13R | agacctctttccaaacttacc | 60 | 154 | |  |  |  |  |
| **STCH** | QSTCH_chr5 | tcctcgtatctcacctccttttcc | QSTCH_chr6 | gcccacctttatgcttcttctcc | 60 | 195 | |  |  |  |  |
| **SAMSN1** | QSAMSN_chr1 | tgtatgaaatcaccttacctttgg | QSAMSN_chr2 | cctgggctcaaccttatctg | 60 | 186 | |  |  |  |  |
| **GAPDH** | QGAPDH-3 | ccatctcagtcgttcccaaagtc | QGAPDH-4 | gccagtcccagcccaagg | 68 | 294 | |  |  |  |  |
| **JB 10** | JB10-563 | gtttcccaccagccaaatgag | JB10-564 | gggcagcacgcagatacc | 60 | 184 | |  |  |  |  |
